# Competitive Pasture Species to Suppress the Growth of Annual Riceflower (*Pimelea trichostachya* Lindl.) at Different Planting Densities and Spatial Arrangements

**DOI:** 10.3390/plants14010082

**Published:** 2024-12-30

**Authors:** Rashid Saleem, Ali Bajwa, Shane Campbell, Mary T. Fletcher, Sundaravelpandian Kalaipandian, Steve W. Adkins

**Affiliations:** 1 School of Agriculture and Food Sustainability, The University of Queensland, Gatton, QLD 4343, Australia; shane.campbell@uq.edu.au (S.C.); s.adkins@uq.edu.au (S.W.A.); 2 La Trobe Institute of Sustainable Agriculture and Food (LISAF), Department of Ecological, Plant and Animal Sciences, AgriBio, La Trobe University, Melbourne, VIC 3086, Australia; a.bajwa@latrobe.edu.au; 3 Queensland Alliance for Agriculture and Food Innovation, The University of Queensland, Coopers Plains, QLD 4108, Australia; mary.fletcher@uq.edu.au; 4 Department of Bioengineering, Saveetha Institute of Medical and Technical Sciences (SIMATS), Saveetha School of Engineering, Chennai 602105, Tamil Nadu, India

**Keywords:** *Pimelea* management, plant competition, perennial pastures, Rhodes grass, cultural weed control

## Abstract

This study assessed the effectiveness of four competitive pasture species—Premier digit grass (*Digitaria eriantha* Steud. var. Premier), Rhodes grass (*Chloris gayana* Kunth.), sabi grass (*Urochloa mosambicensis* Hack.), and buffel grass (*Pennisetum ciliare* L.) against the toxic annual riceflower (*Pimelea trichostachya* Lindl.) at varying planting densities and ratios. At six plants pot^−1^, with a 66:33 grass-to-weed ratio, riceflower biomass decreased by 73.7%, 82.5%, 73.7%, and 60.6% when grown alongside Premier digit, Rhodes, sabi, and buffel grasses, respectively. Similarly, with four plants pot^−1^ at a 75:25 ratio, reductions were 69.1%, 79.8%, 71.0%, and 44.5%, respectively. Annual riceflower experienced the greatest suppression when grown with Rhodes grass, showing aggressivity index (AI) values of −60.2 and −67.2 and relative crowding coefficient (RCC) values of 0.4 for both six and four plants pot^−1^. Premier digit grass also suppressed riceflower effectively, with riceflower AI values of −35.6 and −36.7 and RCC values of 0.5 and 0.6. Buffel grass had the least impact, with riceflower AI values of −41.1 and −27.9 and RCC values of 0.9 and 2.0. Sabi grass also demonstrated good suppressive effects, though slightly less than the top two species. Higher planting densities generally resulted in stronger riceflower suppression. The results highlight the importance of considering planting density, arrangement, and key plant traits when selecting pasture species for successful weed control. Based on these findings, we conclude that Premier digit grass and Rhodes grass show promising potential for effective suppression of annual riceflower growth.

## 1. Introduction

*Pimelea* poisoning, caused by the consumption of annual riceflower (*Pimelea trichostachya* Lindl.) tissues, is one the major causes of economic loss to the livestock industry in Australia [1]. Annual riceflower tissues contain the toxin simplexin which can cause cattle (*Bos taurus* and *B. indicus*) death or the weakening of surviving animals, and a reduction in the stock-carrying capacity of the infested pastures. Annual riceflower is, as the name suggests, an annual, native plant [2]. The nature of the poisoning problem is seasonal, occurring typically during the spring to early summer, but the risk remains present throughout the year due to the toxic nature of the dead plant debris [3]. It is a challenge to manage annual riceflower due to its episodic emergence in the field. Chemical management using herbicides is an effective and efficient management method. However, since the weed can emerge over a vast landscape, chemical control is not economically feasible. It has been proposed that ecological growth suppression using competitive pasture grasses could be a viable alternative and synergetic approach to reduce annual riceflower seedling establishment and growth. When applying any form of management for annual riceflower, it is imperative that the method should not leave any dead plant materials in the field as such tissues remain toxic until naturally dispersed, and could be accidentally consumed by grazing cattle [4].

The use of suppressive pasture plants on several weed species has been explored in Australia. For example, several pasture species were tested against parthenium weed (*Parthenium hysterophorus* L.) and found to be suppressive [5], while producing significant palatable biomass for the livestock grazing those paddocks (the term used for agricultural fields in Australia). In these earlier studies, the suppressive ability of a test species was found to be associated with growth traits, such as rapid height attainment, biomass production, and profuse branching/tillering ability [6,7]. Further, a correlation has been observed between the branching/tillering rate and suppressive ability. The number of tillers increased at the lower plant population densities due to lower shading from the established plants [8]. Pimentel [9] reported that soil resource uptake and light interception ability could be enhanced when the number of tillers is higher. Studies on root traits, such as root number, length, and elongation rate, have shown that roots play a significant role in plant–plant interactions and contribute to suppressive outcomes. This has been highlighted in research by Fargione and Tilman (2006) [10], who found that these root characteristics are critical in determining the overall suppressive impact one plant has on another. Creeping blue grass (*Bothriochloa insculpta* Kuntze) and butterfly pea (*Clitoria ternatea* L.), are thought to suppress parthenium weed growth due to their rapid growth rates, which exceeded that of parthenium weed [11].

The assessment of weed suppression by useful plants can be conducted in a number of ways, but normally through the determination of indices such as the relative crowding coefficient (RCC) [12] and the aggressivity index (AI; [13,14]. The RCC allows for the evaluation and comparison of the suppressive ability (due to competition and allelopathy [5]) of one species against another [15]. In the assessment of pasture species that have weed-suppressing ability, it is best to screen initially under protected conditions in a glasshouse using a pot trial, prior to testing the most promising species under field trials. De Wit (1960) [12] introduced the relative crowding coefficient (RCC or K) in plant competition theory. It gives a measure of the relative dominance of one species over the other in multiple cropping [16].

To date, a study examining the ability of pasture species as suppressive plants against annual riceflower growth has not been undertaken. Hence, this study aims to identify promising pasture species that may have the ability to suppress the growth of annual riceflower under glasshouse conditions. The aim of this study is to explore the potential of hospital paddocks in facilitating the growth and management of pasture species under controlled conditions. In Australia, “hospital paddocks” refer to specific areas of pasture or land designated for the temporary accommodation and care of sick or injured livestock. Specifically, the study seeks to evaluate the effectiveness of four selected nutritious pasture species in suppressing the growth of riceflower. Additionally, it aims to investigate the impact of planting density, arrangement, and key plant traits on determining which pasture species possess the most effective suppressive abilities. By identifying these factors, the study aims to optimize pasture management practices and enhance the control of undesirable riceflower growth.

## 2. Results

### 2.1. Premier Digit Grass

**Figure 1:**
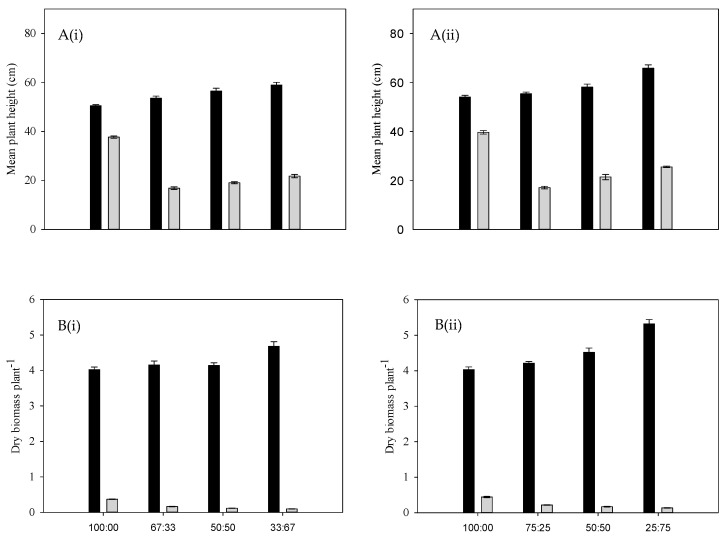
(**A**) Mean plant height of Premier digit grass (black bars) and annual riceflower (grey bars), (**B**) mean dry biomass of Premier digit grass (black bars) and annual riceflower (grey bars), either for the (**i**) 6 or (**ii**) 4 plant pot^−1^ densities, and the 4 planting ratios, assessed 45 days after transplanting. Error bars represent ±2 standard errors of the mean for 10 replicated pots and from 2 repeated experiments.

Annual riceflower grew taller in pure stands of Premier digit grass, with a height attainment of 25.9 cm at a density of four plants pot^−1^, and a height attainment of 23.9 cm at a density of six plants pot^−1^ (Figure 1A).

**Figure 2:**
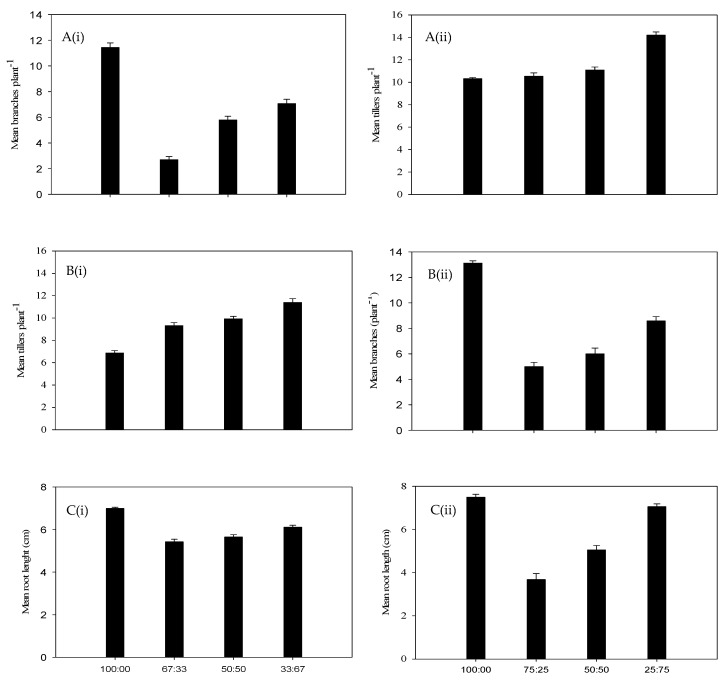
(**A**) Number of tillers plant^−1^ of Premier digit grass (black bars), (**B**) number of branches plant^−1^ of annual riceflower and (black bars), (**C**) mean root length of annual riceflower, either for the (**i**) 6 or (**ii**) 4 plant pot^−1^ densities, and the 4 planting ratios (black bars), assessed 45 days after transplanting. Error bars represent ±2 standard errors of the mean for 10 replicate pots and from 2 repeated experiments.

As the density of Premier digit grass increased, the height attained by annual riceflower progressively decreased (Figure 1A). Similarly, the dry biomass of annual riceflower was significantly reduced by higher densities of Premier digit grass, with the greatest reduction (49.4%) at six plants pot^−1^, as compared to 45.4% at four plants pot^−1^. The lowest biomass was observed at a planting ratio of two annual riceflower to four Premier digit grass plants (Figure 1B, Table 1). In terms of branching, annual riceflower produced the most branches in pure stands, with the highest number (8.2) recorded at four plants pot^−1^, with fewer branches as the density of Premier digit grass increased (Figure 2B). Root length was also affected, with the shortest root length (7.5 cm) observed at a 3:1 ratio of Premier digit grass to annual riceflower (Figure 2C). Overall, the results showed that annual riceflower growth and biomass accumulation were reduced most under the higher densities of Premier digit grass.

### 2.2. Rhodes Grass

**Figure 3:**
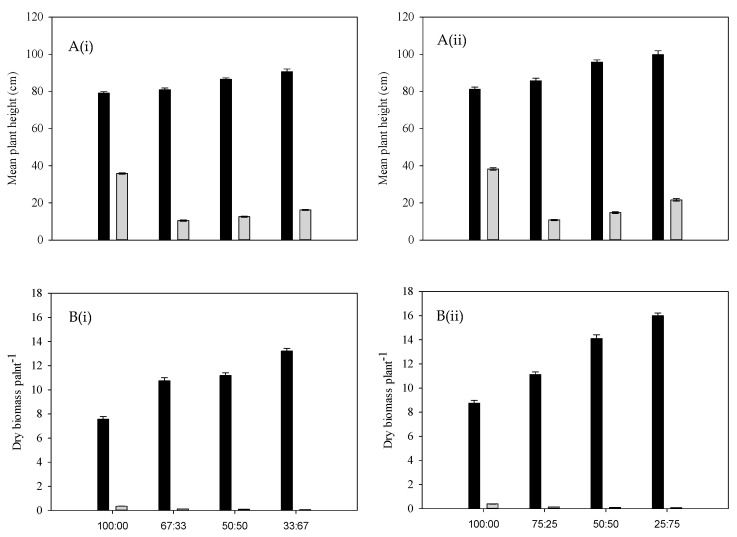
(**A**) Mean plant height of Rhodes grass (black bars), and annual riceflower (grey bars), (**B**) mean dry biomass of Rhodes grass (black bars) and annual riceflower (grey bars), either for the (**i**) 6 or (**ii**) 4 plant pot^−1^ densities, and 4 four planting ratios, assessed 45 days after transplanting. Error bars represent ±2 standard errors of the mean for 10 replicate pots and from 2 repeated experiments.

As the density of Rhodes grass increased, annual riceflower height and dry biomass significantly decreased (*p* < 0.05). The lowest annual riceflower height was found at the ratio of one annual riceflower plant to three Rhodes grass plants, at the four plants pot^−1^ density (Figure 3A). The tallest annual riceflower plants were at the four plants pot^−1^ density (Figure 3A) compared to six plants pot^−1^ (18.8 cm), with the maximum height in pure stands. (Figure 3A). Dry biomass of annual riceflower was lowest at two annual riceflower plants to four Rhodes grass plants at six plants pot^−1^ density, while it was highest in pure stands (Figure 3B, Table 1). The highest biomass reduction was at six plants pot^−1^ density (82.5%) compared to four plants pot^−1^ density (79.8%). The lowest dry biomass was found at the ratio of two annual riceflower plants (33%) to four Rhodes grass plants (67%) at six plants pot^−1^ density, and the highest was in in pure stands (Figure 3B, Table 1).

**Figure 4:**
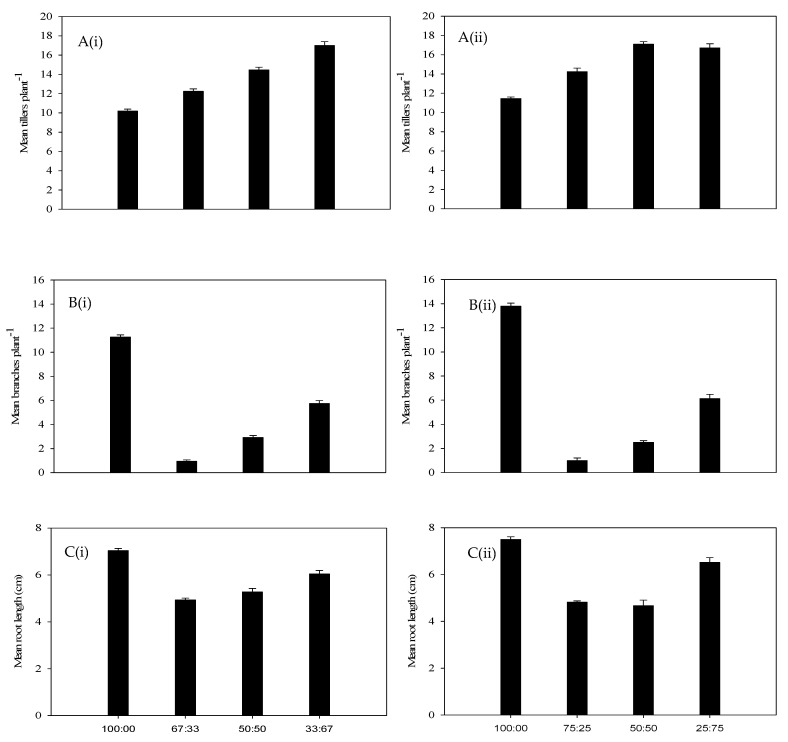
(**A**) Number of tillers plant^−1^ of Rhodes grass, (**B**) number of branches plant^−1^ of annual riceflower, and (**C**) mean root length of annual riceflower, either for the (**i**) 6 or (**ii**) 4 plant pot^−1^ densities, and 4 planting ratios, assessed 45 days after transplanting. Error bars represent ±2 standard errors of the mean for 10 replicate pots and from 2 repeated experiments.

The highest number of branches (5.9) was at four plants pot^−1^ density compared to six plants pot^−1^ density (5.2) (Figure 4B). The root length of annual riceflower was highest in pure stands at four plants pot^−1^ density (7.4 cm) and at six plants pot^−1^ density (7.0 cm). The lowest root length (6.6 cm) was found at the 50:50 ratio (Rhodes grass: annual riceflower) at four plants pot^−1^ density, and (6.9 cm) at the 67:33 ratio in six plants pot^−1^ (Figure 4C).

### 2.3. Sabi Grass

**Figure 5:**
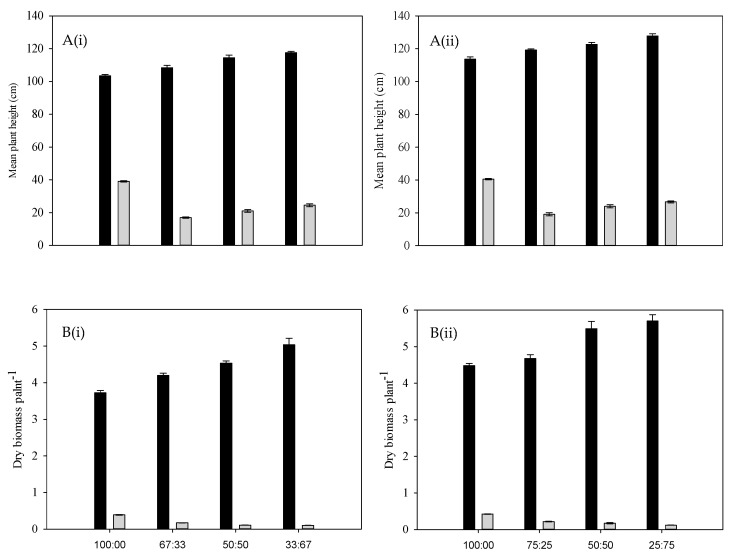
(**A**) Mean plant height of sabi grass (black bars) and annual riceflower (grey bars), (**B**) mean dry biomass of sabi grass (black bars) and annual riceflower (grey bars), either for the (**i**) 6 or (**ii**) 4 plant pot^−1^ densities, and the 4 planting ratios, assessed 45 days after transplanting. Error bars represent ±2 standard errors of the mean for 10 replicate pots and from 2 repeated experiments.

**Figure 6:**
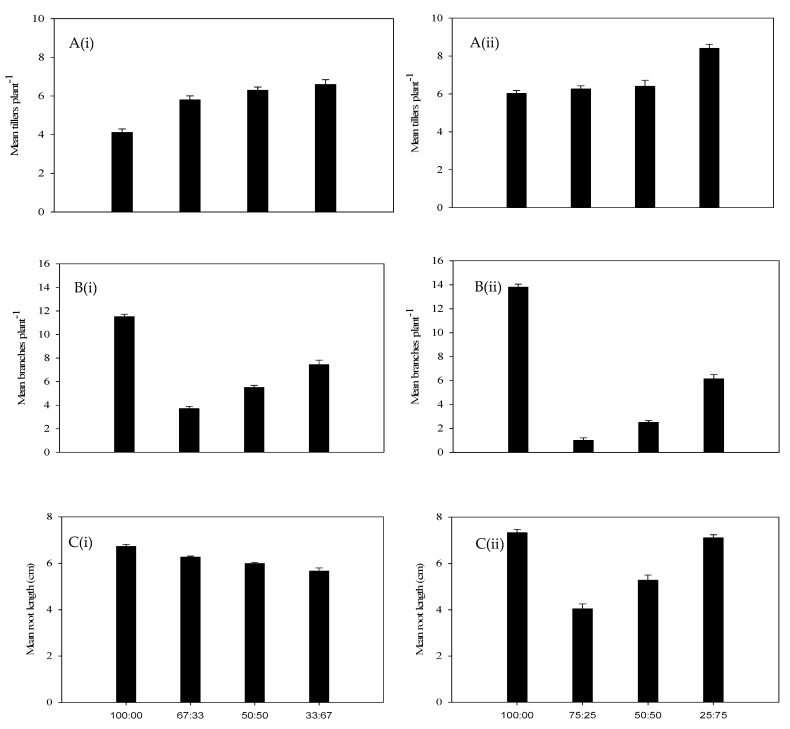
(**A**) Number of tillers plant^−1^ of sabi grass, (**B**) number of branches plant^−1^ of annual riceflower, and (**C**) mean root length of annual riceflower, either for the (**i**) 6 or (**ii**) 4 plant pot^−1^ densities, and 4 planting ratios, assessed 45 days after transplanting. Error bars represent ±2 standard errors of the mean for 10 replicate pots and from 2 repeated experiments.

The plant height of annual riceflower was significantly (*p* < 0.05) influenced by planting density and ratios. The shortest annual riceflower plants (25.0%) were observed at the ratios of one annual riceflower plant to three sabi grass plants at four plants pot^−1^ density (Figure 5A), while the tallest (27.5 cm) were at the four plants pot^−1^ density (Figure 6A). In contrast, sabi grass height was affected by both planting density and ratios, with the highest height at one annual riceflower plant (75%) to one sabi grass plant at four plants pot^−1^ density (Figure 5A).

The dry biomass of sabi grass significantly increased (17.5%) at four plants pot^−1^ density, whereas the annual riceflower biomass decreased with increasing sabi grass density, remarkably at six plants pot^−1^ density. The highest reduction in annual riceflower biomass (73.7%) occurred at the ratios of two annual riceflower plants (33%) to four sabi grass plants at six plants pot^−1^ density (67.0%), with the highest annual riceflower biomass at the ratios of four annual riceflower plants (100%) to zero sabi grass plants per pot^−1^ (Figure 5B; Table 1). The number of sabi grass tillers was significantly influenced by planting ratios, with the highest number (7.8) at the ratios of 1:3 (sabi grass: annual riceflower) at four plants pot^−1^ density (Figure 6A). In contrast, annual riceflower had the highest number of branches (9.1) at four plants pot^−1^ density and the highest root length (7.3 cm) in pure stands at four plants pot^−1^ density (Figure 6B,C). Overall, the results indicated an intricate relationship between planting density and ratios in shaping the growth and biomass production of annual riceflower and sabi grass.

### 2.4. Buffel Grass

**Figure 7:**
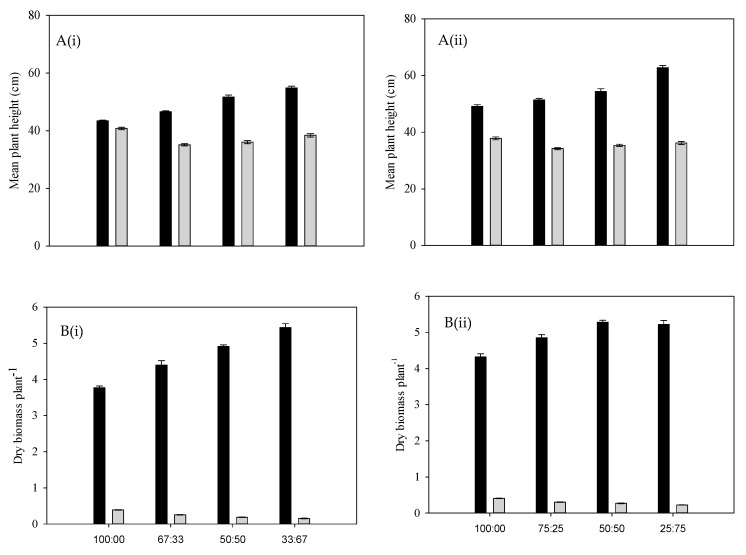
(**A**) Mean plant height of buffel grass (black bars) and annual riceflower (grey bars), (**B**) mean dry biomass of buffel grass (black bars) and annual riceflower (grey bars), either for the (**i**) 6 or (**ii**) 4 plant pot^−1^ densities, and the 4 planting ratios and assessed 45 days after transplanting. Error bars represent ±2 standard errors of the mean for 10 replicate pots and from 2 repeated experiments.

The plant height of annual riceflower was significantly (*p* < 0.05) affected by planting density and ratios. The shortest plants (25.0%) were observed at a ratio of one plant to three buffel grass plants at four plants pot^−1^ density (75%), while the tallest (27.5 cm) were observed at the four plants pot^−1^ density (Figure 7A). The plants grew taller at the four plants pot^−1^ density (37.6 cm) compared to six plants pot^−1^ density (35.9 cm).

**Figure 8:**
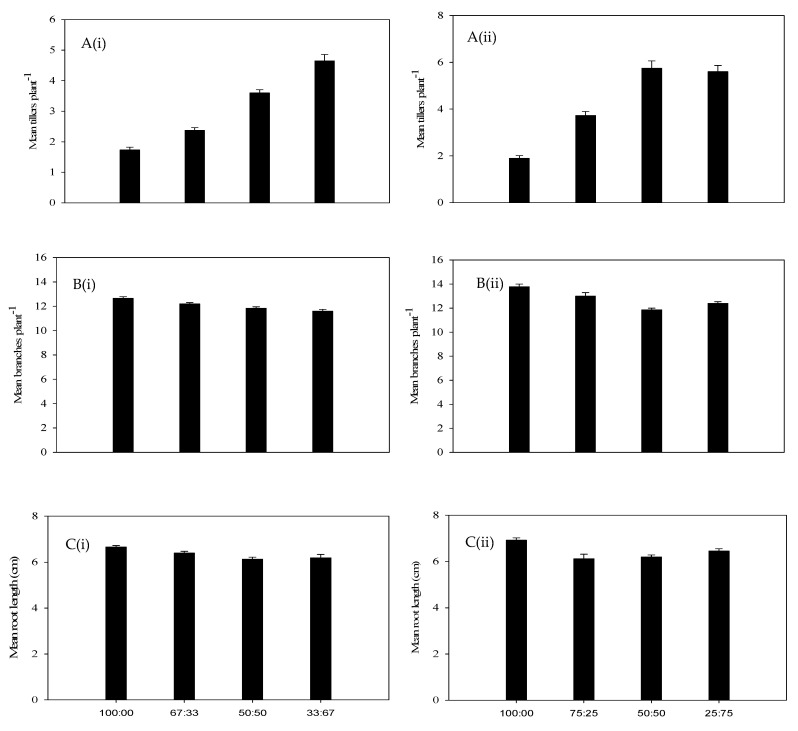
(**A**) Number of tillers plant^−1^ of buffel grass, (**B**) number of branches plant^−1^ of annual riceflower, and (**C**) mean root length of annual riceflower, either for the (**i**) 6 or (**ii**) 4 plant pot^−1^ densities, and 4 planting ratios, assessed 45 days after transplanting. Error bars represent ±2 standard errors of the mean for 10 replicate pots and from 2 repeated experiments.

The highest biomass reduction of annual riceflower was recorded at the six plants pot^−1^ density (36.8%) compared to the four plants pot^−1^ density (25.8%). The lowest annual riceflower dry biomass (60.6%) was observed at a ratio of two annual riceflower plants (33.0%) to four buffel grass plants at the six plants pot^−1^ density (67.0%); (Figure 7B, Table 1), while the highest dry biomass was recorded at a ratio of four annual riceflower plants (100%) to zero buffel grass plants per pot^−1^ (Figure 7B, Table 1). The dry biomass of buffel grass was significantly (*p* < 0.05) affected by planting density and ratios, showing the highest increase (22.7%) at the four plants pot^−1^ density (Figure 7B). The number of tillers and branches of annual riceflower were significantly (*p* < 0.05) affected by planting density and ratios, with the highest values observed at the four plants pot^−1^ density and in pure stands, respectively (Figure 8A,B). The root length of annual riceflower was significantly (*p* < 0.05) affected by planting ratios, with the lowest recorded at a ratio of 75:25 (buffel grass: annual riceflower) at the four plants pot^−1^ density (Figure 8C). The highest root length (6.9 cm) of annual riceflower was recorded in pure stands at the four plants pot^−1^ density.

### 2.5. Relative Crowding Coefficients and Aggressivity Index for Grass Species

The Table 2 presents the relative crowding coefficient (RCC) and aggressivity index (AI) values for Premier digit grass, Rhodes grass, sabi grass, buffel grass, and annual riceflower grown together at a planting ratio of 50:50 in two different planting densities: six plants per pot (six plant pot^−1^) and four plants per pot (four plant pot^−1^). At six plants pot^−1^, Premier digit grass exhibited an RCC of −33.5, indicating strong intra-specific competition, while Rhodes grass showed an RCC of −3.1, similarly indicating competitive ability within its own species. Sabi grass had an RCC of −5.6, and buffel grass had an RCC of −4.3, both indicating strong competition within their species as well. In contrast, annual riceflower had a positive RCC of 0.5 with Premier digit grass, 0.4 with Rhodes grass, 0.4 with Sabi grass, and 0.9 with Buffel grass, indicating weaker competitive ability compared to the grass species.

At four plants per pot, the RCC values shifted slightly, with Premier digit grass having an RCC of −9.3, Rhodes grass −2.6, sabi grass −5.4, and buffel grass −5.5. Annual riceflower maintained similar RCC values with Premier digit grass (0.6), Rhodes grass (0.4), sabi grass (0.7), and buffel grass (2.0), indicating persistent weaker competitive ability compared to the grass species. The aggressivity index (AI) values at six plants pot^−1^ were as follows: Premier digit grass (35.6), Rhodes grass (60.2), sabi grass (46.9), buffel grass (41.1), and annual riceflower (−35.6 to −46.9). At four plants pot^−1^, the AI values were Premier digit grass (36.7), Rhodes grass (67.2), Sabi grass (40.5), Buffel grass (27.9), and annual riceflower (−36.7 to −27.9). These values indicate that Premier digit grass, Rhodes grass, sabi grass, and buffel grass are more competitively dominant over annual riceflower, as evidenced by their negative RCC and higher positive AI values, which suggest a greater ability to suppress annual riceflower dry biomass. Moreover, the differences between the four and six plants pot^−1^ densities showed slight variations in competitive abilities, with generally higher competition observed at the higher planting density of six plants pot^−1^.

## 3. Discussion

Pasture cropping had a significant suppression effect on weed density and diversity, and this can be used as a valid weed management tool [17]. The suppressive effect of pasture cropping on weeds could be related to its early competition for resources such as nutrients and light [18]. This study aimed to assess four selected, nutritious pasture species for their ability to supress annual riceflower growth and to determine the effect of planting density and ratios on the weed growth. We also identified the important plant traits which are associated with the suppressive ability. In this study, Premier digit and Rhodes grass species were best in supressing weed growth, although the suppression mechanism varied. For example, the high tillering capacity of Premier digit grass (Figure 2A) and vigorous growth, and tillering of Rhodes (Figure 4A) grass may be attributed to the suppression of annual riceflower. This suppression was verified by RCC and AI values depicting strong interplant competition (Table 2). Premier digit grass, Rhodes grass, and sabi grass were observed to grow significantly taller than annual riceflower, while buffel grass grew only marginally taller than it. Shabbir et al. [19] also reported that introduced and native pasture plant species can suppress the growth of parthenium weed. Khan [20] reported rapid plant height attainment to be an important attribute that helps plants to capture light and can therefore act as a good indicator of a plant’s suppressive ability. The height attained by these three pasture species (Premier digit grass, Rhodes grass, and sabi grass) was rapid, and significantly reduced the plant height of annual riceflower, especially in the Spring conditions when annual riceflower grew at its best (Figure 1A, Figure 3A and Figure 5A). Overall, the height of annual riceflower progressively decreased as the plant density of grass species increased, while the height attainment of grass species was reduced as the plant density of grass species increased. Long-term weed suppression can be achieved by using different cultural methods, including the selection of adequate planting density in combination with environmental conditions [21]. Riceflower height was drastically decreased, indicating that grass species were able to suppress and apply pressure to the growth of annual riceflower. This might be due to the shading effect and high tillering capacity of pasture species. Walk et al. [22] reported that early leaf expansion can enhance the suppressive ability of plants by promoting shading on the neighbouring plant community. Shade discourages the growth and establishment of all three species in the *P. simplex* group [3]. Plants growing in competition also suppress growth, with reported observations of wild parsnip (*Trachymene* spp.) suppressing the growth of *P. elongata* [3]. However, with decreasing population density of pasture species, the plant height of annual riceflower was improved. Plant species with appropriate growth traits (viz. efficient nutrient uptake, higher water use efficiency adaptation to a wide range of climates, and having an extensive rooting system and a large leaf canopy) can suppress the growth of weeds [23]. Test species with greater height, speedy tiller production, or a branched canopy and root system strongly suppressed the growth of *Parthenium hysterophorus* L. [5]. In this study, all of the tested grass species suppressed the annual riceflower growth, but the exact suppression mechanism of these species is unknown as we did not conduct any biochemical analysis. However, suppression could be associated with their physical (plant competition) and/or chemical (allelopathy) attributes [24]. The suppressive effect of a species denotes the capability to which it can supress the growth of neighbouring plants by competing for resources [25] and/or by allelopathy. Competition between plants is mainly for light, water, nitrogen, and other minerals, and the rate of uptake of these resources plays a significant role in plant growth [26]. The pasture competition trials indicated that the pasture species studied had a strong ability to suppress the growth and biomass of annual riceflower. Several factors can affect the growth of a species when grown in a mixture, particularly the planting ratios, the spatial ratios, plant density, cultivar planted, and the suppressive ability (defined as a combination of a plant’s competitive and allelopathic ability) [5] between the mixture components [27,28]. Reductions in biomass are observed in mixtures when inter-specific suppression is higher than intra-specific suppression [15].

The shorter buffel grass was found least effective in suppressing annual riceflower growth under the test environments (Figure 7A). Earlier studies suggested that annual riceflower can grow well in dense buffel grass pasture [3] and it was also validated through famer’s survey that overgrazed pastures encourage the abundance of annual riceflower. Scanty pastures usually encourage germination and seedling establishment due to low competition. For instance, Mitchell grass and wiregrass offer least competition during early phase of growth and development but start competition in later stage due to intense growth [3]. Despite a plant being suppressive in a set of controlled environmental conditions may have different its suppressive ability in the field [29] due to the biotic and abiotic factors which could greatly influence growth performance of plants. Different plant attribute, for instance, early emergence and root elongation [30], penetration ability into the soil and root branching trait [31]. Premier digit, Rhodes, Sabi, and Buffel grasses all demonstrate strong competitiveness against annual riceflower, as evidenced by the significant reductions in annual riceflower biomass across different planting ratios and densities. Premier digit grass and Rhodes grass showed the highest reductions in annual riceflower biomass, with reductions of up to 49.4% to 82.5% at six plants per pot. Sabi grass also exhibited significant biomass reduction (up to 73.7%) at the same planting density. Buffel grass, while showing slightly lower reductions (up to 36.8%), still effectively suppressed annual riceflower growth (Table 1). This suggests that these grass species are capable of outcompeting and suppressing annual riceflower growth when grown together. Negative RCC values for the grasses suggest strong intra-specific competition, while higher positive AI values indicate their greater ability to suppress dry biomass of annual riceflower. Generally, higher densities and specific planting ratios (favouring more grass plants relative to annual riceflower) result in greater reductions in annual riceflower biomass. This indicates that higher grass densities can enhance their competitive advantage and reduce the growth of annual riceflower more effectively. This is consistent with the growth pattern of pasture species, which had more suppressing ability under higher population densities. Synergistic interactions may occur among species due to modifications in the resource utilization. Relative species abundances also determine the strength of an interaction. These ideas can be applied to assess the yield and plant suppression of multispecies grasslands [32], and to develop ideal pasture [33].

Annual riceflower branches were reduced either with increasing plant population or increased tillers (Premier digit grass, Rhodes grass, and sabi grass) per unit area (Figure 2B, Figure 4B and Figure 6B). A greater number of branches or tillers enhance a plant’s ability to capture light and to promote their resource capture capabilities [20]. However, it also possible that during an active tillering phase, a plant’s suppressive ability may decline due to the redirection of energy resources from height attainment to branching [20]. All the pasture species and their planting density influenced the number of branches produced by annual riceflower. In addition, the number of branches produced by annual riceflower was greater in the in the four-plant density than in the six-plants density, indicating that the test species have more competitive ability when present in higher densities.

Relative crowding coefficients (RCC) were calculated for annual riceflower and grass species when grown together. The RCC values for annual riceflower and grass species were positive and negative, respectively under all combinations used in this study. The RCC values indicate the competitive interactions between the grasses and annual riceflower, with negative values showing suppression of annual riceflower by the grasses (Table 2). Premier digit, Rhodes, Sabi, and Buffel grasses all exhibit negative RCC values, suggesting they suppressed annual riceflower growth. The AI values indicate the dominance of the grasses over annual riceflower, with positive values indicating greater competitiveness. All the four grasses had positive AI values, confirming their dominance over annual riceflower. However, the inter-specific competitiveness of grass species is reduced with the increasing density of grass plants in each pot, indicating that inter-plant competition of grass species weakens the inter-specific competitiveness of grass when calculated at an individual plant level (per plant basis). Reductions in yield are observed in mixtures when inter-specific suppression is higher than intra-specific suppression [15]. These findings underscore their potential use in managing pastures and highlight the importance of understanding species interactions for effective pasture management strategies.

## 4. Materials and Methods

### 4.1. Pasture Species

Four perennial forage grass species [Rhodes grass (*Chloris gayana* Kunth.), buffel grass (*Pennisetum ciliare* L.), sabi grass (*Urochloa mosambicensis* Hack.), and Premier digit grass (*Digitaria eriantha* Steud.); Table 3] were selected for study based on their growth habits and vigorous vegetative growth. These species were selected after consulting a number of pasture management specialists and reviewing the available literature. The trial was carried out during 2019 and repeated during 2020 at the University of Queensland (UQ), Gatton Campus, Australia.

Before testing the grass species in a field where riceflower grows, it is important to determine how a weed might respond to competition from grasses with different growth habits, as this will aid selection for competitive species for field testing.

### 4.2. Annual Riceflower Seed Collection, Processing and Storage

Annual riceflower plants bearing mature and immature fruits were hand-collected from an oat (*Avena sativa* L.) cultivation paddock in Maranoa Shire, (83.611093061° S, 159.419863512° E; elevation of 257 m) at a location 76 km south of the town of Roma, Queensland, during September 2018. The plants were typically multi-branched, 30 to 40 cm tall, and were growing in a red sandy soil. One of the collected plants was sent as a voucher specimen to the Queensland herbarium (BRI number AQ1010277) and later confirmed as annual riceflower. The remaining plants were sun-dried for 2 weeks, and then the mature fruits were gently removed by shaking onto a paper sheet. The fruits were again sun-dried for a further week, then placed into cotton cloth bags and kept under laboratory conditions (22 ± 2 °C) to attain an air-dried condition. The cleaned single-seeded fruits were then placed into labelled paper bags and stored in airtight plastic containers in the dark at 15 ± 2 °C temperature and 15 ± 5% relative humidity in a seed store at the UQ Gatton Campus.

### 4.3. Plant Preparation

Seed lots of the four pasture grass species were obtained from Toowoomba (Queensland, Australia). These seed lots were stored until required in a seed store at a constant 15 ± 2 °C temperature, with a 15 ± 5% relative humidity. Seeds of the four test species were taken from the seed storage, and in lots of 100 seeds, spread over moistened paper towels within sealed plastic containers (30 × 20 × 10 cm; length × width × height). The containers were placed into a greenhouse until sufficient germination had occurred, approximately 1 week later for Premier digit grass and Rhodes grass and 2 weeks later for buffel grass and sabi grass.

Annual riceflower fruit (here after referred to as seeds) were scarified and soaked in 1.15 mM GA_3_ for 24 h before sowing. The primed annual riceflower seeds were then sown individually into water-saturated Jiffy pellets (Jiffy-7 42 mm single pellet, Jiffy Products International, AS, Stange, Norway), held in a plastic tray, and then placed into the greenhouse to obtain seedlings for experimentation. After 15 to 20 days, the emergence of annual riceflower seedlings commenced and continued for 1 week, until sufficient seedlings of both annual riceflower and pasture grass species (now ca. 2 cm tall) were produced. The soil was provided by a local farmer whose paddock was heavily infested with *Pimelea*, making it an ideal resource for a plant competition study. Healthy and uniform-sized seedlings were then selected and transplanted into 25 cm diameter plastic pots containing ca. 7.5 L of soil that had been previously watered to field capacity (soil had been saturated with tap water and allowed to freely drain for 24 h). The densities of seedlings set up were either four or six plants per pot. The Premier digit grass and Rhodes grass trials were carried out on two occasions (1 month apart), with each trial being run for 45 days after transplantation in the greenhouse during the spring months of September to October 2019. The sabi and buffel grass trials were also carried out on two occasions (1 month apart) and run for 45 days after transplantation during September to October 2020.

### 4.4. Measurements

The experimental setup involved randomly distributing the pots across benches within a greenhouse situated at The University of Queensland’s Gatton Campus. This approach was crucial to minimize any systematic effects of environmental variations within the poly tunnel on the experimental outcomes. Additionally, the plants were watered at regular intervals using a Bluetooth-supported irrigation system, which allowed precise control over both the timing and frequency of irrigation. This advanced system was programmed to deliver water at predetermined times, ensuring consistent soil moisture levels critical for the growth and competition dynamics of the plants. All plants were evaluated 45 days after transplantation with plant height (measured from soil level to tip of tallest plant part) and the number of branches or tillers produced by each plant determined. Shoots were clipped at soil level, placed individually into brown paper bags, dried in an oven at 65 ± 2 °C for 48 h, and then dry biomass was recorded. For root measurements, the roots were carefully washed free of soil and blotted dry. Root length was measured. In addition, calculations were made of the relative crowding coefficient (RCC) and aggressivity index (AI), as described below. The RCC (a measure of the relative dominance of one species over the other [12]) was determined as follows:

K_grass =_ DMYgp × Zpg/((DMYg − DMYgp) × Zgp),

K_pimelea =_ DMYpg × Zgp/((DMYp − DMYpg) × Zpg),

where DMYgp and DMYpg are the dry matter yields of the grass (g) and *Pimelea* (p) when grown in combinations, respectively. DMYg and DMYp are the dry matter yields of the grass and the *Pimelea* in the control (no other species present), respectively. Zgp is the ratio of grass and Zpg is the ratio of the *Pimelea* planting. A negative value for K_gp_ indicates a strong suppressiveness of the grass species g, while a positive value demonstrates a weak suppressiveness. This means that the magnitude of K_gp_ indicates the strength of the suppressive ability of the grass species. An illustration of all the possible combinations of K_gp_ values for both g and p species are shown in Table 4.

The aggressivity index (AI) measures how much the relative yield of one plant component is greater than that of another plant [13].

Aggressivity is expressed as:

AI_grass_ = (DMYgp/DMYg × Zpg) − (DMYpg/DMYp × Zgp)

AI_pimelea_ = (DMYpg/DMYp × Zgp) − (DMYgp/DMYg × Zpg)

If AI_grass_ or AI_pimelea_ = 0, both crops are equally competitive. When AI_grass_ is positive then the grass species is dominant, and when it is negative then *Pimelea* is the dominating species.

For each of the four test species and annual riceflower shoot, biomass reduction was expressed as a percentage of dry shoot [38] using the following modified equation.

Biomass reduction (%) = (C − B)/C × 100

where C is the dry shoot biomass in the control treatment (no other species present) and B is the shoot biomass of the average plant biomass of an individual experimental unit. The data analysis of parameters was undertaken in two steps, (1) involving the effect of increasing planting ratios of pasture grass species (0, 25, 50, and 75% for four plants, and 0, 33, 50, and 67% for six plants) on annual riceflower growth, and (2) the effect of increasing ratios of annual riceflower (0, 25, 50, and 75% for four plants, and 0, 33, 50, and 67% for six plants) against the test species.

### 4.5. Data Analysis

The data were averaged from the two repeated experiments and then analysed, including two densities and respective plant ratios. All data sets were subjected to ANOVA using the general linear model (GLM) with a split plot design in Statistix 8.1. Planting densities were assigned to the main plots, while planting ratios were assigned to the subplots. SigmaPlot 14.0 was used to graphically present the treatment means, with standard error bars added to represent the variability and uncertainty in the data.

## 5. Conclusions

This study highlights the potential of utilizing competitive pasture species as a sustainable and effective approach to managing toxic plants, such as annual riceflower. By strategically selecting and planting species like Rhodes grass, Premier digit grass, and Sabi grass at optimal densities and ratios, land managers can enhance pasture health, reduce the reliance on chemical weed control, and improve overall land sustainability. This approach aligns with the growing emphasis on ecological solutions in land management, promoting more environmentally friendly practices. The findings suggest a promising direction for improving pasture resilience and supporting long-term, sustainable grazing systems. These results also suggest that hospital paddocks, which provide controlled conditions for growing pasture species, can play a crucial role in managing annual riceflower growth in grazing lands. By employing these ecological strategies, land managers can strengthen the suppressive abilities of competitive pasture species, leading to healthier pastures and more sustainable grazing systems.

## Figures and Tables

**Table 1:** Percent decrease in dry biomass, assessed 45 days after transplanting, of the 4 test plant species and annual riceflower when grown in at either a 6 or 4 plants pot^−1^ planting density, in the 4 planting ratios.

**Planting Density**	**Six Plants Pot^−1^** **Percent Decrease in Dry Biomass (g)**								
Test plant to ARF *	Premier digit	ARF	Rhodes	ARF	Sabi	ARF	Buffel	ARF	Mean
66:33	16.3	73.7	74.9	82.5	35.3	73.7	44.1	60.6	57.7
50:50	3.1	68.2	48.0	72.4	21.8	71.9	30.2	52.0	46.0
33:66	3.3	55.9	42.3	62.7	12.7	55.5	16.7	34.6	35.5
Mean test plants	5.7		41.3		17.5		22.7		
Mean ARF		49.5		54.4		50.3		36.8	
**Planting Density**	**Four Plants Pot^−1^** **Percent Decrease in Dry Biomass (g)**								
Test plant to ARF	Premier digit	ARF	Rhodes	ARF	Sabi	ARF	Buffel	ARF	Mean
75:25	31.8	69.1	82.9	79.8	27.2	71.0	20.8	44.5	53.4
50:50	12.02	61.5	61.2	73.2	22.5	58.5	22.2	33.0	43.1
25:75	4.4	50.8	27.0	63.5	4.3	48.3	12.2	25.4	29.5
Mean test plants	12.0		42.8		13.5		13.8		
ARF		45.3		54.1		44.4		25.8	

Annual riceflower (ARF) *.

**Table 2:** Relative crowding coefficient (RCC) and aggressivity index (AI) for 4 test species grown with annual riceflower at a planting ratio of d50:50 plants, in a density of either 6 or 4 plants pot^−1^.

Test Species	Six Plant Pot^−1^		Four Plant Pot^−1^	
Grass or *Pimelea*	RCC	AI	RCC	AI
Premier digit	−33.5	35.6	−9.3	36.7
Annual riceflower	0.5	−35.6	0.6	−36.7
Rhodes	−3.1	60.2	−2.6	67.2
Annual riceflower	0.4	−60.2	0.4	−67.2
sabi	−5.6	46.9	−5.4	40.5
Annual riceflower	0.4	−46.9	0.7	−40.5
buffel	−4.3	41.1	−5.5	27.9
Annual riceflower	0.9	−41.1	2.0	−27.9

**Table 3:** A description of the perennial pasture species used in the study to assess their suppressive ability against annual riceflower.

Species	Brief Description and Reasons for Selection
Premier digit grass (*Digiteria eriantha* Steud.)	Premier digit grass (*Digitaria eriantha* spp. *eriantha*) is a perennial summer-growing grass. It is adapted to low fertility on granite, sandstone, and traprock soils of South East Queensland (SEQ) and the more fertile, scrub, and lighter clay soils of the Darling Downs, the Maranoa and Western Downs. It is not suitable for heavy alkaline clay soils. Premier digit grass grows well at minimum of 600 mm annual rainfall (Department of Agriculture and Fisheries, Queensland,).
Rhodes grass (*Chloris gayana* Kunth.)	Rhodes grass (*Chloris gayana* Kunth.) has become one of the major forage grasses planted throughout the tropical and sub-tropical regions of the globe and can be grazed with moderate to high feed quality [34]. Rhodes grass flourishes in areas with an annual rainfall of 600 to 1600 mm. The grass can be grown on a varied range of soils, from clays to sandy loams. It grows well under irrigation and is moderately tolerant to flooding. The grass is palatable to animals with good nutritive value, especially in the early growth stages [35].
Sabi grass (*Urochloa mosambicensis* Hack.)	*Urochloa mosambicensis* (Hack.) Dandy, commonly known as sabi grass, is a robust perennial grass with a creeping, stoloniferous growth pattern. It is widely utilized in southern Africa for various purposes, including as livestock forage, a cereal crop, soil erosion prevention, and the rehabilitation of degraded areas such as mine sites. Renowned for its rapid growth, drought and salinity resistance, adaptability to diverse soils and fertility levels, tolerance to heavy grazing, and efficient seed production, sabi grass stands out as a promising pasture grass for semiarid tropical regions [36].
Buffel grass (*Pennisetum ciliare* L.)	Buffel grass (*Pennisetum ciliare* L.) is extensively cultivated across tropical and subtropical arid rangelands worldwide due to its exceptional drought tolerance and resilience under heavy grazing. However, beyond its natural habitat, it can aggressively spread into native ecosystems, roadsides, and urban areas, disrupting wildfire regimes and outcompeting native plant and animal species [37].

**Table 4:** Possible combinations of relative crowding coefficient values of species g and p when grown in combination, and its explanation.

Species g (Grass)	Species p (*Pimelea*)	Meaning
+K_gp_ (K_gp_ > K_pg_)	+K_pg_	Both species exhibit weak inter-specific competitiveness, but species g is stronger than species p
–K_gp_ (K_gp_ > K_pg_)	0 –K_pg_	Both species exhibit strong inter-specific competitiveness, but species p is stronger than species g
+K_gp_	–K_pg_	Species g exhibits weak inter-specific competitiveness, species p is competitively strong in inter-specific interactions
K_gp_ = 0	K_pg_ = 0	Both species do not affect each other
K_gp_ = 0	+K_pg_	Species g does not affect species p, while the latter exhibits weak inter-specific competitiveness
K_gp_ = 0	–K_pg_	Species g does not affect species p, while the latter exhibits strong inter-specific competitiveness

## Data Availability

Data are contained within the article.
